# Ca(H_2_PO_4_)_2_ and MgSO_4_ activated nitrogen-related bacteria and genes in thermophilic stage of compost

**DOI:** 10.1007/s00253-024-13167-6

**Published:** 2024-05-11

**Authors:** Lihong Jiang, Jiapeng Dai, Lutong Wang, Liang Chen, Guangxi Zeng, Erlun Liu, Xiangdan Zhou, Hao Yao, Yunhua Xiao, Jun Fang

**Affiliations:** 1 College of Bioscience and Biotechnology, Hunan Agricultural University, Changsha, 410128 China; 2 Hunan Engineering Laboratory for Pollution Control and Waste, Utilization in Swine Production, Changsha, 410128 China; 3 Board of Directors Department, Changsha IMADEK Intelligent Technology Company Limited, Changsha, 410137 China

**Keywords:** Ca(H_2_PO_4_)_2_, MgSO_4_, Activate related bacteria, Nitrogen metabolism genes, Compost product fertility

## Abstract

**Abstract:**

This study was conducted to investigate the effects of Ca(H_2_PO_4_)_2_ and MgSO_4_ on the bacterial community and nitrogen metabolism genes in the aerobic composting of pig manure. The experimental treatments were set up as control (C), 1% Ca(H_2_PO_4_)_2_ + 2% MgSO_4_ (CaPM1), and 1.5% Ca(H_2_PO_4_)_2_ + 3% MgSO_4_ (CaPM2), which were used at the end of composting for potting trials. The results showed that Ca(H_2_PO_4_)_2_ and MgSO_4_ played an excellent role in retaining nitrogen and increasing the alkali-hydrolyzed nitrogen (AN), available phosphorus (AP), and available potassium (AK) contents of the composts. Adding Ca(H_2_PO_4_)_2_ and MgSO_4_ changed the microbial community structure of the compost. The microorganisms associated with nitrogen retention were activated. The complexity of the microbial network was enhanced. Genetic prediction analysis showed that the addition of Ca(H_2_PO_4_)_2_ and MgSO_4_ reduced the accumulation of nitroso-nitrogen and the process of denitrification. At the same time, despite the reduction of genes related to nitrogen fixation, the conversion of ammonia to nitrogenous organic compounds was promoted and the stability of nitrogen was increased. Mantel test analysis showed that Ca(H_2_PO_4_)_2_ and MgSO_4_ can affect nitrogen transformation-related bacteria and thus indirectly affect nitrogen metabolism genes by influencing the temperature, pH, and organic matter (OM) of the compost and also directly affected nitrogen metabolism genes through PO_4_^3−^ and Mg^2+^. The pot experiment showed that composting with 1.5% Ca(H_2_PO_4_)_2_ + 3% MgSO_4_ produced the compost product that improved the growth yield and nutrient content of cilantro and increased the fertility of the soil. In conclusion, Ca(H_2_PO_4_)_2_ and MgSO_4_ reduces the loss of nitrogen from compost, activates nitrogen-related bacteria and genes in the thermophilic phase of composting, and improves the fertilizer efficiency of compost products.

**Key points:**

*• Ca(H*_*2*_ *PO*_*4*_ *)*_*2*_ *and MgSO*_*4*_ *reduced the nitrogen loss and improved the compost effect*

*• Activated nitrogen-related bacteria and altered nitrogen metabolism genes*

*• Improved the yield and quality of cilantro and fertility of soil*

## Introduction

The rapid development of livestock farming and agriculture has produced much agricultural waste, including livestock manure and crop straw (Mengqi et al. 2021). Swine manure is one of the major livestock manures, and up to 2.03 billion metric tons of swine manure is produced by swine farming each year in China (Zhou et al. 2023). Pig manure contain a large amount of organic matter, which can be reused as resources, but they also contain many harmful substances, such as heavy metals, antibiotics, and pathogenic bacteria (Wu et al. 2023). These agricultural wastes can cause damage to the environment if they are casually discarded or discharged without treatment. Therefore, it is especially urgent to find an environmentally friendly way to dispose of these agricultural wastes.

Aerobic compost is an efficient, fast, and environment-friendly way to treat organic waste. Aerobic composting uses microorganisms widely existing in nature to promote the biochemical process of converting degradable organic matter in organic waste into stable humus (Guo et al. 2018). The microorganisms in the aerobic composting process can passivate heavy metals and also eliminate antibiotics (Zhang et al. 2019). Pig manure can be turned into a soil amendment and fertilizer for crops by aerobic composting, making it a practical approach to sustainable waste management and organic farming. Compost products were utilized in agricultural production to improve soil quality and crop yields (Pergola et al. 2018). For example, compost products were considered to improve maize yield and soil fertility and is a low-pollution risk fertilizer (He et al. 2022). Compost products could promote soil organic carbon sequestration by regulating the conversion of soil permanganate oxidizable carbon fraction (He et al. 2023). Compost products could increase soil organic matter, total nitrogen, and available phosphorus content (Casado-Vela et al. 2007). The study found that the compost products converted from straw compost (wheat straw and rice straw) contains many trace elements like Zn, Mg, and Fe and had many multi-trait bacterial consortium AAP (*Azospirillum*, *Arthrobacter*, and *Pseudomonas* spp.), and it can significantly promote the growth and yield of pepper and tomato (Imran et al. 2021).

The quality and efficiency of composting were often improved by adding various additives. At present, there are three main types of compost additives: (1) physical additives: materials with physical adsorption properties such as zeolite and biochar are used to reduce nitrogen loss in compost (Liao et al. 2021; Manu et al. 2022); (2) biological additives: these mainly consist of the addition of exogenous microorganisms to improve composting efficiency and maturity (Jiang et al. 2015); and (3) chemical additives: mainly through the addition of chemical reagents to change the internal properties of compost to achieve excellent composting effect, such as magnesium chloride and ferrous sulfate (Li et al. 2020c). Ca(H_2_PO_4_)_2_ and MgSO_4_ as chemical additives have stable effects and low cost compared to physical and biological additives. Adding phosphate can promote the temperature increase and organic matter degradation in composting (Li et al. 2020a). Meanwhile, adding phosphate could reduce the loss of nitrogen during composting (Wang et al. 2019) and introduce other nutrients to improve compost quality (Yuan et al. 2018). For example, phosphate can increase the content of total and available phosphorus in the compost (Sarr et al. 2020). In addition, MgSO_4_ is an additive that can promote nitrogen retention and decay of compost. Adding MgSO_4_ and KH_2_PO_4_-K_2_HPO_4_ effectively maintained the pH range and retained more ammonia in the compost to promote struvite (MgNH_4_PO_4_·6H_2_O) formation (Liang et al. 2018). In composting, PO_4_^3−^ and Mg^2+^ formed MgHPO_4_ in equal amount and then further combined with NH_4_^+^ to form struvite, which achieved the effect of nitrogen retention in composting (Jiang et al. 2016). The struvite formation has a higher reaction rate and further promotes ammonia fixation when the Mg^2+^ content is higher than PO_4_^3−^ (Huang et al. 2014), and ratio of PO_4_^3−^ and Mg^2+^ at 1:2 is better for nitrogen retention than at 1:1 (Wang et al. 2013).

PO_4_^3−^ and Mg^2+^ can retain nitrogen either through their specific properties or by modifying the internal physicochemical properties of the compost. However, the bacterial community and nitrogen metabolism genes in the composting process have not been studied in sufficient depth. Moreover, the effectiveness of composting for practical applications can be further verified. The study was conducted to investigate the aerobic composting of pig manure by co-addition different amounts of Ca(H_2_PO_4_)_2_ and MgSO_4_ based on the better ratio of molar amounts of PO_4_^3−^ and Mg^2+^ (1:2) for nitrogen retention and to further investigate the bacterial community and nitrogen metabolism genes of the compost.

## Materials and methods

### Raw materials and experimental design

Composting was carried out in the Cultivation Garden shed at Hunan Agricultural University, Changsha, Hunan, China. Pig manure was provided by Hanshou Pig Farm (Changde, China), and straw was collected from suburban farmland (Changsha, China). Pig manure was a fresh sample. Rice straw was dried and chopped into 1 ~ 2 cm pieces. The physicochemical properties of pig manure and rice straw are shown in Table 1. Ca(H_2_PO_4_)_2_ and MgSO_4_ were purchased from Beijing Solarbio Science & Technology Limited Company, Beijing, China. The C/N ratio of pig manure was adjusted to 25 with rice straw, and then, the moisture content was adjusted to 60%. Three treatments were observed: control (C, without Ca(H_2_PO_4_)_2_ and MgSO_4_), 1% Ca(H_2_PO_4_)_2_ + 2% MgSO_4_ (CaPM1), and 1.5% Ca(H_2_PO_4_)_2_ + 3% MgSO_4_ (CaPM2). The ratio of PO_4_^3−^ and Mg^2+^ added to CaPM1 and CaPM2 was about 1:2 (*n*/*n*). Three replicates were set up for each treatment. The composting experiment was conducted in 100 L PVC containers for 30 days. The compost was turned once every 2 days to maintain aerobic conditions. Samples to be analyzed were obtained at 10 d and 30 d using five-point sampling method. The samples were divided into two parts: samples for determination of physical and chemical properties were stored at 4 ℃ for backup, and samples for microbiological data were stored at − 80 °C for storage. The organic fertilizer obtained from each treatment group was added to the soil for the pot experiment to verify the fertility effect of organic fertilizer. Each treatment group was mixed with soil according to the additional amount of 1% organic fertilizer, 800 g per pot of fertilizer soil mixture (Liu et al. 2021). The cilantro (*Coriandrum sativum* L.) used in the pot experiment needs to be raised in advance and then transplanted. Three pots were planted in each treatment, three plants per pot, and CK was treated with equal amounts of soil not mixed with organic fertilizer. The potted cilantro was harvested 45 days after planting, and the collected samples were temporarily stored in the 4 °C refrigerator.

**Table 1:** Physical and chemical properties of raw materials

Raw materials	Moisture (%)	Total organic carbon (g/kg)	Total nitrogen (g/kg)	C/N
Pig manure	34.89 ± 1.31	393.96 ± 3.23	19.99 ± 1.22	19.71 ± 1.58
Rice straw	16.37 ± 1.22	583.87 ± 4.21	9.84 ± 0.89	59.34 ± 2.98

Note: each indicator was measured three times for each sample

### Physicochemical analysis

The water extracts of pH were extracted by 1:10 (w/v) of sample and water (Wang et al. 2021). The pH measured with a pH meter (PSB-25, Laici, China). The total organic carbon (TOC) content of raw materials was determined by K_2_Cr_2_O_7_ volumetric method (Qu et al. 2022). Organic matter (OM) and total nitrogen (TN) were quantified by K_2_Cr_2_O_7_ titration and Kjeldahl nitrogen determination (Qian et al. 2018). After the sample was digested by H_2_SO_4_-HClO_4_ method, the molybdenum-antimony anti-colorimetric method was used to determine the total phosphorus (TP), and a flame photometer determined the total potassium (TK). Determination of alkali-hydrolyzed nitrogen (AN) content by an alkaline hydrolysis diffusion method. The available phosphorus (AP) and available potassium (AK) were extracted with NaHCO_3_ and CH_3_COONH_4_, respectively. The AP extract was determined by the molybdenum-antimony anti-colorimetric method, and the AK extract was determined by flame photometry. Pakchoi (*Brassica campestris* L. ssp. *Chinensis Makino*) seeds were used for the germination index (GI) measurement. The GI was determined in accordance with the method of (Kong et al. 2022) and calculated with the following equation:

$$\mathrm{GI}=\frac{\mathrm{number}\;\mathrm{of}\;\mathrm{germinated}\;\mathrm{seeds}\;\mathrm{in}\;\mathrm{extract}}{\mathrm{number}\;\mathrm{of}\;\mathrm{germinated}\;\mathrm{seeds}\;\mathrm{in}\;\mathrm{control}}\times\frac{\mathrm{root}\;\mathrm{length}\;\mathrm{in}\;\mathrm{extract}}{\mathrm{root}\;\mathrm{length}\;\mathrm{in}\;\mathrm{control}}\times100\%$$

Cilantro samples were measured for plant height and root length. Chlorophyll content was measured using a chlorophyll meter (JC-YLS01, Juchuang, China). Cilantro weight by balance, cilantro water content by balance method, root viability coefficient by TTC method, vitamin C by DCPIP titration, soluble sugars by anthrone method, soluble protein by Coomassie Brilliant Blue G-250 method were observed.

### DNA extraction and high-throughput sequencing

Genomic DNA was extracted from compost samples using a DNA kit (MN NucleoSpin 96 Soil, TIANGEN, China). Bacterial 16S rRNA (V3 + V4) region was amplified using universal PCR primers 338F: 5′-ACTCCTACGGGAGGCAGCA-3′, 806R: 5′-GGACTACHVGGGTWTCTAAT-3′. The final products were sequenced on Illumina NovaSeq 6000 platform. FLASH (version 1.2.11) splices the raw data, Trimmomatic (version 0.33) quality filters the spliced sequences, and removes chimeras with UCHIME (version 8.1) to obtain high-quality Tags sequences (Magoč and Salzberg 2011; Bolger et al. 2014). USEARCH (version 10.0) classified clean sequences into operational taxonomic units (OTUs) with a similarity cut-off value of 97% (Edgar 2013). The RDP Classifier (version 2.2, http://sourceforge.net/projects/rdpclassifier/) was used to classify and annotate representative sequences of bacterial OTUs on the Silva databases, respectively (Wang et al. 2007; Kõljalg et al. 2013). The original reads generated in the study have been submitted to NCBI’s SRA, accession: PRJNA936923.

### Statistical analysis and microbial community and functional analysis

All experiments were replicated in triplicates, and general statistical analysis was performed by Excel 2016. The statistics of compost and potted Cilantro were carried out by one-way ANOVA utilizing SPSS (version 25.0). The community structure of microorganism was conducted by partial least squares discrimination analysis (PLS-DA), relative abundance histogram, and linear discriminant analysis effect size (LEfSe). The correlation between top fifteen genus and environmental factors was shown by correlation heat map. Data processing for network analysis was done by R (version 4.1.0), and network plotting was done by Gephi (version 0.9.7). Presentation of bacterial community gene prediction data was done using the LianChuan BioCloud platform (https://www.omicstudio.cn/tool) to produce volcano maps, KEGG pathway color maps, and mantel test analysis.

## Results

### The physicochemical properties of compost

**Fig. 1:**
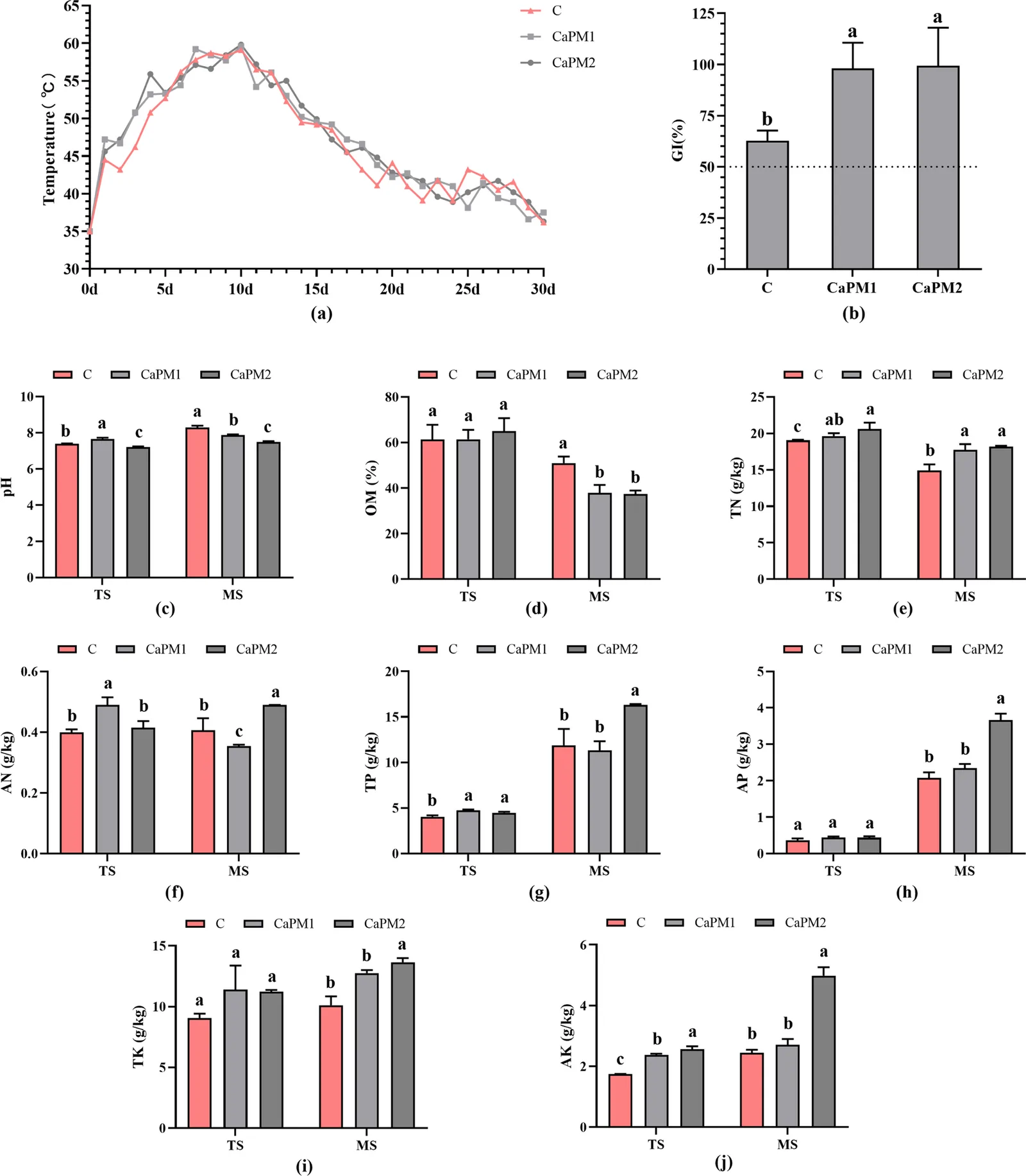
Physicochemical indexes of composting. **a** Temperature. **b** Germination index. **c** pH. **d** Organic matter. **e** Total nitrogen. **f** Alkali-hydrolyzed nitrogen. **g** Total phosphorus. **h** Available phosphorus. **i** Total potassium. **j** Available potassium. In the figure, TS indicates the thermophilic stage, and MS indicates the mature stage. C: control (without Ca(H_2_PO_4_)_2_ and MgSO_4_); CaPM1: 1% Ca(H_2_PO_4_)_2_ + 2% MgSO_4_; CaPM2: 1.5% Ca(H_2_PO_4_)_2_ + 3% MgSO_4_. The letters above the bars indicate significant differences at *p* < 0.05

The physicochemical properties of the composting process were shown in Fig. 1. The composting temperature gradually increased and peaked at 10 d of composting with 59.1 °C, 59.6 °C, and 59.8 °C for C, CaPM1, and CaPM2, respectively (Fig. 1a). All three groups had germination index (GI) values above 50% at the end of composting, and the GI values of CaPM1 and CaPM2 were significantly higher than the GI values of C (*p* < 0.05) (Fig. 1b). Figure 1c was clear that the pH values for the thermophilic phase (TS) and the maturity phase (MS) of the compost were in the range of 7.21 ~ 7.40 and 7.49 ~ 8.30 for each treatment. The pH of TS was slightly higher than that of MS, and both periods showed weak alkalinity. After the composting process, the organic matter (OM) content in the MS showed a decreasing trend compared to the TS (Fig. 1d). The total nitrogen (TN) loss during TS to MS was 9.72% and 11.75% for CaPM1 and CaPM2, respectively, both smaller than 21.62% for C, and the TN content of CaPM1 and CaPM2 in MS was significantly higher than that of C (*p* < 0.05) (Fig. 1e). TN loss was less in both CaPM1 and CaPM2 than in C during TS to MS. This result indicates that adding Ca(H_2_PO_4_)_2_ and MgSO_4_ in composting can reduce nitrogen loss, achieve the effect of nitrogen retention, reduce nutrient loss, and maintain high organic fertilizer efficiency. The CaPM2 treatment group consistently had the highest level of alkali-hydrolyzed nitrogen (AN) during the MS (Fig. 1f). All composts showed an increase in total phosphorus (TP) and total potassium (TK) content from the TS to MS (Fig. 1g, i). The AP content of each treatment group did not differ significantly during the high-temperature period. However, available phosphorus (AP) increased in all treatment groups at the MS, with CaPM1 and CaPM2 increasing by 429.89% and 733.09%, respectively. CaPM2 has a significantly higher AP content at the MS than the other groups (*p* < 0.05) (Fig. 1h). The available potassium (AK) content in CaPM2 was significantly higher (*p* < 0.05) than in C and CaPM1 during composting (Fig. 1j). As a whole, the CaPM2 treatment group of 1.5% Ca(H_2_PO_4_)_2_ + 3% MgSO_4_ showed better physicochemical properties than the other treatment groups.

### Analysis of bacterial community structure

**Fig. 2:**
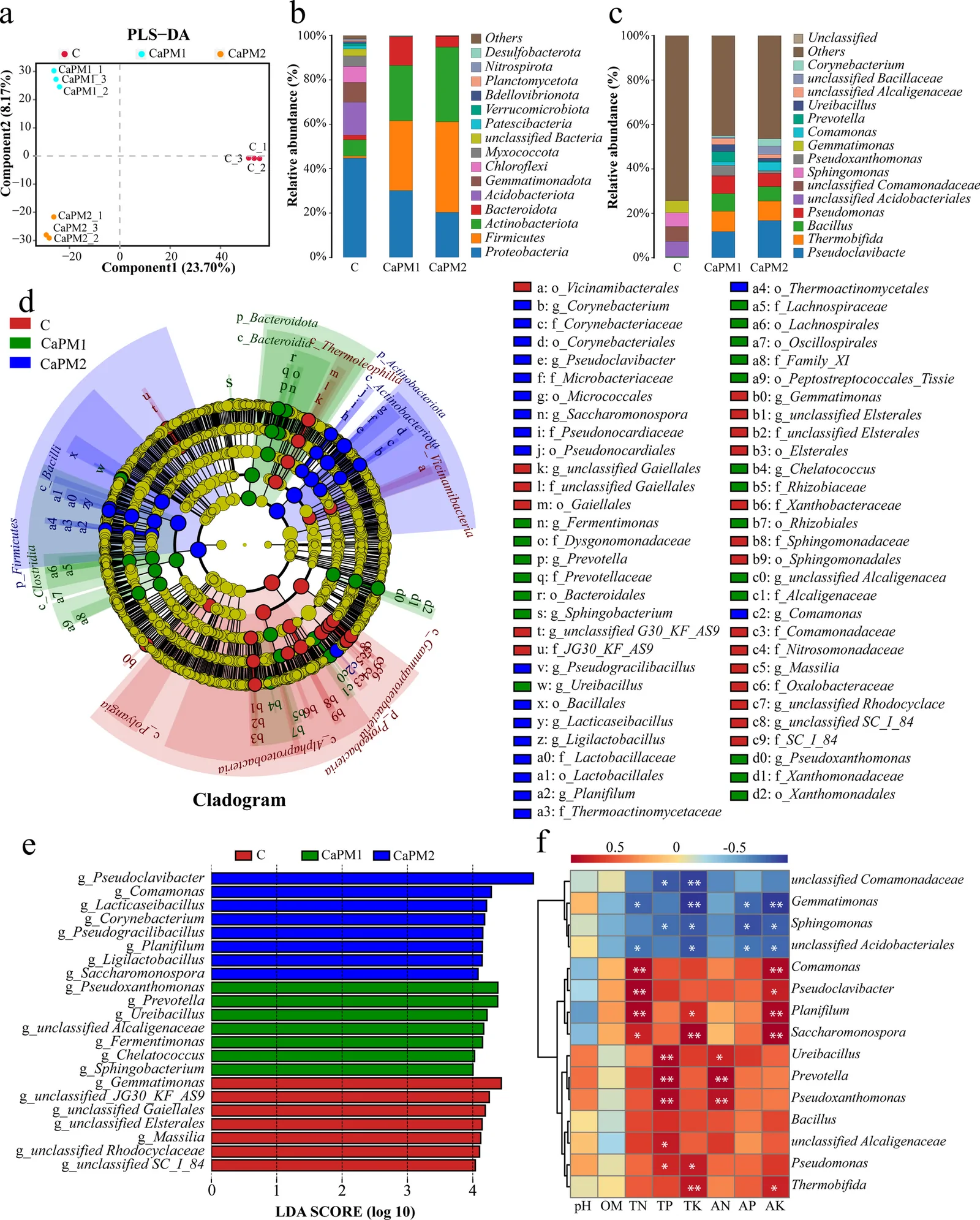
Analysis of the structure, composition, variation and correlation with physicochemical properties of bacterial communities. **a** Partial least squares discriminant analysis (PLS-DA) of bacterial microbial communities based on OTU levels (component1 23.70%, component 2 8.17%). **b** Relative abundance composition of bacterial communities at the phylum level. **c** Relative abundance composition of bacterial communities at the genus level. **d** Linear discriminant analysis effect size (LEfSe) evolutionary branch diagram of bacteria. Circles radiating inward to outward represent taxonomic levels from phylum to species, with every other small circle representing a taxonomy at that level; taxa with no significant differences are uniformly colored yellow, and other differential taxa are colored according to the subgroup with the highest abundance in which the species is found. **e** LDA score distribution histogram of bacterial species level (*p* < 0.05, LDA score > 4). **f** Heat map of the correlation between physicochemical properties of compost and bacteria (genus level). Correlations were obtained by Spearman correlation calculations (*p* < 0.05, *R* > 0.9). In all figures, C: control (without Ca(H_2_PO_4_)_2_ and MgSO_4_); CaPM1: 1% Ca(H_2_PO_4_)_2_ + 2% MgSO_4_; CaPM2: 1.5% Ca(H_2_PO_4_)_2_ + 3% MgSO_4_

As shown in the PLS-DA analysis, C, CaPM1 and CaPM2 were farther apart in the plot, which indicated that the bacterial communities have a large variation (Fig. 2a). As shown in Fig. 2b, in the experimental groups CaPM1 and CaPM2, the main phyla were *Proteobacteria* (30.11% and 20.29%), *Firmicutes* (31.48% and 40.82%), and *Actinobacteria* (24.91% and 33.67%), which is to previous studies (Li et al. 2022; Zhu et al. 2021). However, this is different from the results of Wang et al. (2023a, b), which showed up to 77.46% ~ 92.83% of the *Firmicutes* during composting, a result much higher than the values of the present study. The dominant genera in C were *unclassified Acidobacteriales* (6.96%), *unclassified Comamonadaceae* (6.69%), and *Sphingomonas* (6.23%), but the dominant genera in CaPM1 and CaPM2 were *Pseudoclavibacter* (11.75% and 36.63%), *Thermobifida* (9.19% and 16.7%), and *Bacillus* (7.99% and 8.83%) (Fig. 2c). The LEfSe analysis found statistically different biomarkers among the different groups. The circles inside and outside the evolutionary branch of the LEfSe analysis represent taxonomic levels from phylum to species. The significance of differences among microbial groups in different treatments was analyzed by LEfSe analysis. The LDA score bar shows that different genera of bacteria exist in different treatment groups (*p* < 0.05, LDA score > 4). Different biomarkers in CaPM1 and CaPM2 of the treatment groups with the addition of Ca(H_2_PO_4_)_2_ and MgSO_4_ were 20 and 19, respectively (Fig. 2e).

Among the differences in bacterial genera, the significantly different genera for CaPM1 were *Pseudoxanthomonas*, *Prevotella*, *Ureibacillus*, *Chelatococcus*, *Fermentimonas*, *Sphingobacterium*, and *unclassified Alcaligenaceae*. The genus with significant differences in CaPM2 were *Lacticaseibacillus*, *Planifilum*, *Corynebacterium*, *Ligilactobacillus*, *Comamonas*, *Pseudogracilibacillus*, *Saccharomonospora*, and *Pseudoclavibacter*. The heat map of the relationship between bacteria and physicochemical properties during the thermophilic stages in Fig. 2f showed that TN and AK were significantly positively correlated with *Saccharomonospora*, *Planifilum*, *Pseudoclavibacter*, and *Comamonas* (*p* < 0.05). TP and AN were significantly positively correlated with *Pseudoxanthomonas*, *Prevotella*, and *Ureibacillus* (*p* < 0.05). TK was significantly correlated with *Thermobifida*, *Pseudomonas*, *Saccharomonospora*, and *Planifilum* were significantly positively correlated (*p* < 0.05).

### Co-occurrence network analysis

**Fig. 3:**
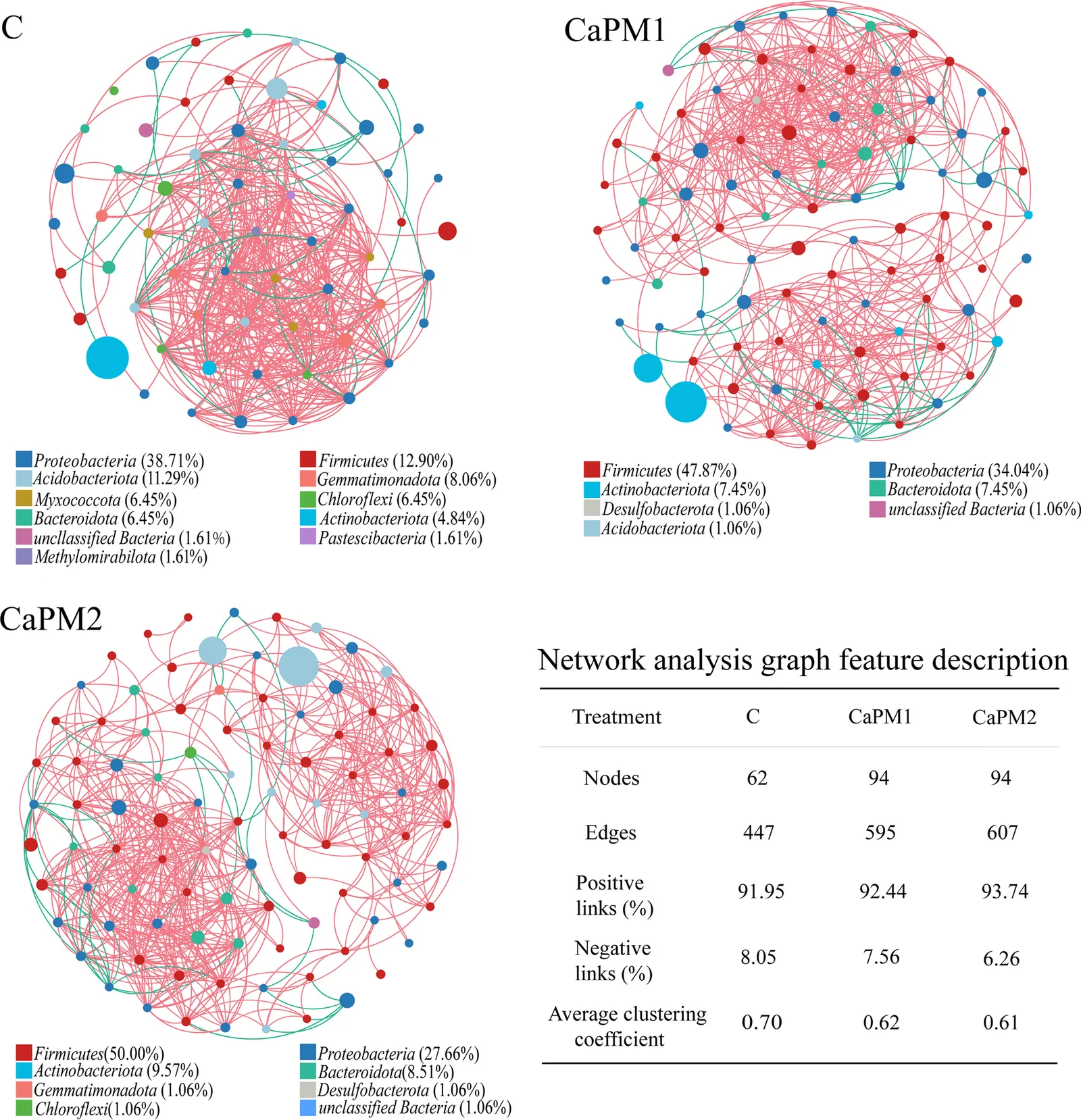
The co-occurrence network visualizing the correlations among species in C, CaPM1, and CaPM2. Correlations were obtained by Spearman correlation calculations (*p* < 0.05, *R* > 0.7), and network analysis graph feature description. Different circles represent different species, the size of the circle represents the abundance of the species, and the color of the circle represents the bacterial phylum to which the species belongs. The color of the edge represents positive and negative correlation, red represents positive correlation, green represents negative correlation. C: control (without Ca(H_2_PO_4_)_2_ and MgSO_4_); CaPM1: 1% Ca(H_2_PO_4_)_2_ + 2% MgSO_4_; CaPM2: 1.5% Ca(H_2_PO_4_)_2_ + 3% MgSO_4_

Network analysis is a crucial way to study microbial community interaction patterns that highlights microbial communities’ characteristics from a network perspective. Figure 3 shows the microbial network analysis of composting bacterial species. Compared to C, the networks of CaPM1 and CaPM2 are more extensive. The results showed that adding Ca(H_2_PO_4_)_2_ and MgSO_4_ increased the proportion of *Firmicutes* in the co-occurrence network while decreasing the proportion of *Proteobacteria*. From network analysis graph feature description, the proportion of positive links in the network edges of C, CaPM1, and CaPM2 were all much greater than the proportion of negative links. The main negative links in C are centered between *Proteobacteria*, *Acidobacteriota*, *Chloroflex*, and *Myxococcota*, whereas the main negative links in CaPM1 and CaPM2 are centered between *Proteobacteria*, *Firmicutes*, and *Acidobacteriota*. *Proteobacteria* and *Acidobacteriota* were involved in most of the negative linkages in all three treatments. The nodes in the networks of C, CaPM1, and CaPM2 are 62, 94, and 94, respectively, and the edges are 447, 595, and 607, respectively. CaPM1 and CaPM2 both had more nodes and edges in the network than C. CaPM1 and CaPM2 both had 94 nodes, but CaPM2 had the most network edges. The addition of Ca(H_2_PO_4_)_2_ and MgSO_4_ to the compost improved the interaction of the compost bacterial community and increased the complexity of the network.

### Changes in abundance of nitrogen metabolism genes

**Fig. 4:**
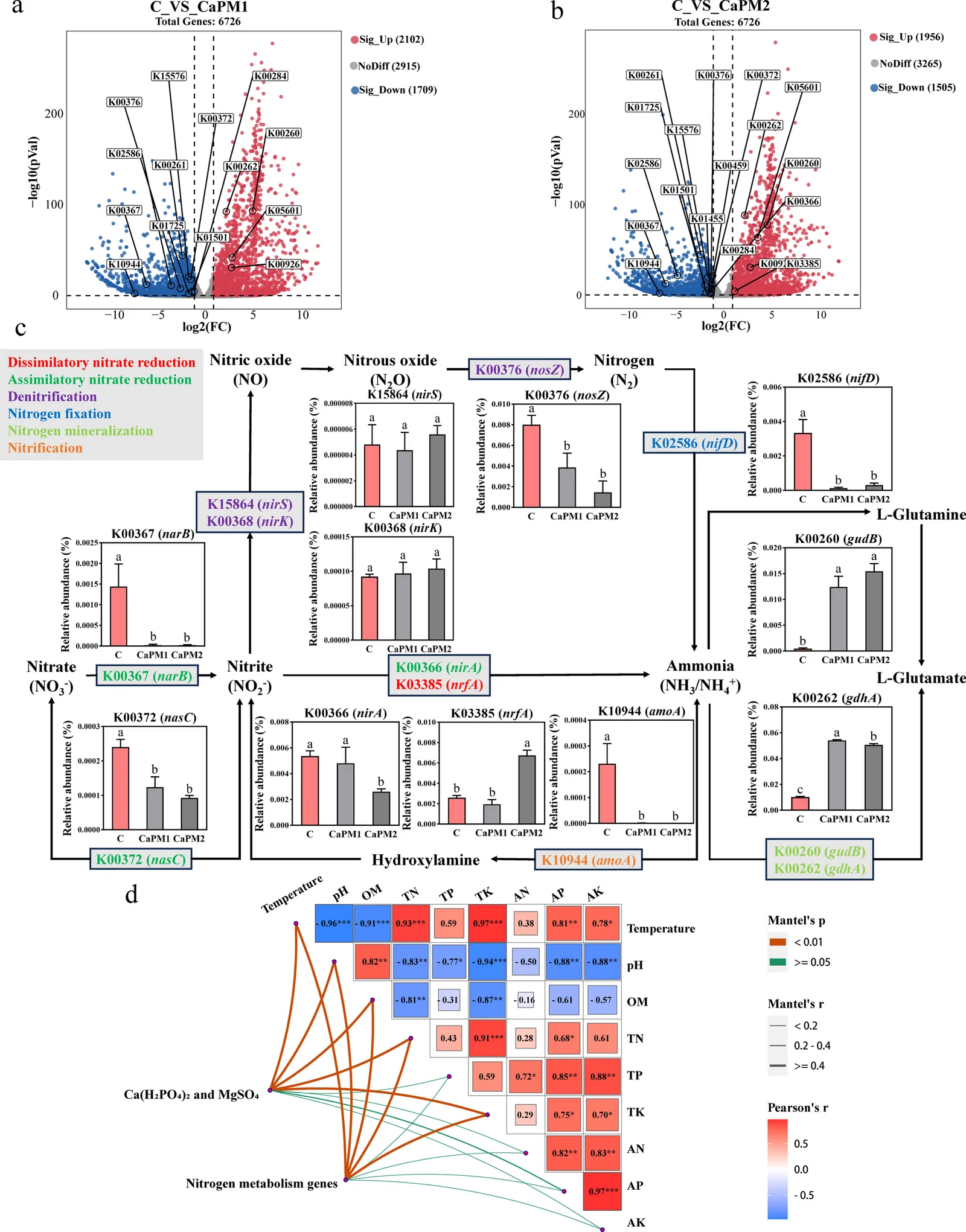
Changes in the abundance of nitrogen metabolism genes. **a** Volcano map of genes differentially expressed in CaPM1 relative to C. **b** Volcano map of genes differentially expressed in CaPM2 relative to C. Each dot in the volcano diagram represents a gene, with red dots being significantly up-regulated genes and blue dots being significantly down-regulated genes. **c** Nitrogen metabolism genes and their corresponding pathways. The different colored genes (names) indicate different metabolic pathways, and the bar graph on the way is the relative abundance of the corresponding genes. The letters above the bars indicate significant differences at *p* < 0.05. **d** Mantel test analysis of the relationship between compost additives and physicochemical properties and nitrogen metabolism genes and physicochemical properties. C: control (without Ca(H_2_PO_4_)_2_ and MgSO_4_); CaPM1: 1% Ca(H_2_PO_4_)_2_ + 2% MgSO_4_; CaPM2: 1.5% Ca(H_2_PO_4_)_2_ + 3% MgSO_4_

To explore the effects of Ca(H_2_PO_4_)_2_ and MgSO_4_ on nitrogen conversion during the thermophilic stage of pig manure composting, the prediction and analysis of related functional gene abundance were carried out using PICRUSt2. The volcano plot shows that both CAPM1 and CAPM2 involved 6726 genes in the analysis (Fig. 4a, b). There were 10 significantly down-regulated and 4 significantly up-regulated nitrogen metabolism genes in CaPM1; 13 significantly down-regulated and 5 significantly up-regulated nitrogen metabolism genes in CaPM1. Genes with significantly up-regulated and significantly down-regulated nitrogen metabolism in CaPM1 and CaPM2 obtained by volcano mapping were labeled in the KEGG pathway color map (Fig. 4c). Nitrogen metabolic processes include nitrogen fixation, assimilatory nitrate reduction, dissimilatory nitrate reduction, denitrification, nitrification, complete nitrification, and Anammox (Li et al. 2021). Compared with C, CaPM1 was down-regulated at K02586 (*nifD*) in nitrogen fixation, K00367 (*narB*), and K00372 (*nasC*) in assimilatory nitrate reduction, K03385 (*nrfA*) in dissimilatory nitrate reduction, and K10944 (*amoA*) in nitrification. K00376 (*nosZ*) in denitrification. K00260 (*gudB*) and K00262 (*gdhA*) in the ammonia and L-glutamate conversion pathways were up-regulated. Compared with C, CaPM2 was down-regulated in K02586 (*nifD*) in nitrogen fixation, K00367 (*narB*), K00372 (*nasC*) and K00366 (*nirA*) in assimilatory nitrate reduction, K00376 (*nosZ*) in denitrification, and K10944 (*amoA*) in nitrification. K03385 (*nrfA*) in dissimilatory nitrate reduction and K00260 (*gudB*) and K00262 (*gdhA*) in the ammonia-L-glutamate conversion pathway are up-regulated. The nitrite reduce coding genes K00368 (*nirK*) and K15864 (*nirS*), which are key genes for denitrification, were not significantly different in all treatment groups. Nitrogen metabolism genes are influenced by environmental factors in the composting, so it is necessary to explore the relationship between nitrogen metabolism genes and physicochemical properties. From the mantel test analysis (Fig. 4d), it can be seen that the indicators that were significantly correlated with Ca(H_2_PO_4_)_2_ and MgSO_4_ were temperature, pH, OM, TN, and TK (*p* < 0.01). Nitrogen metabolism genes were also significantly correlated with temperature, pH, OM, TN, and TK (*p* < 0.01). The correlations between compost additives and compost physicochemical properties and the correlations between nitrogen metabolism genes and compost physicochemical properties were highly similar.

### Coriander pot experiment

**Fig. 5:**
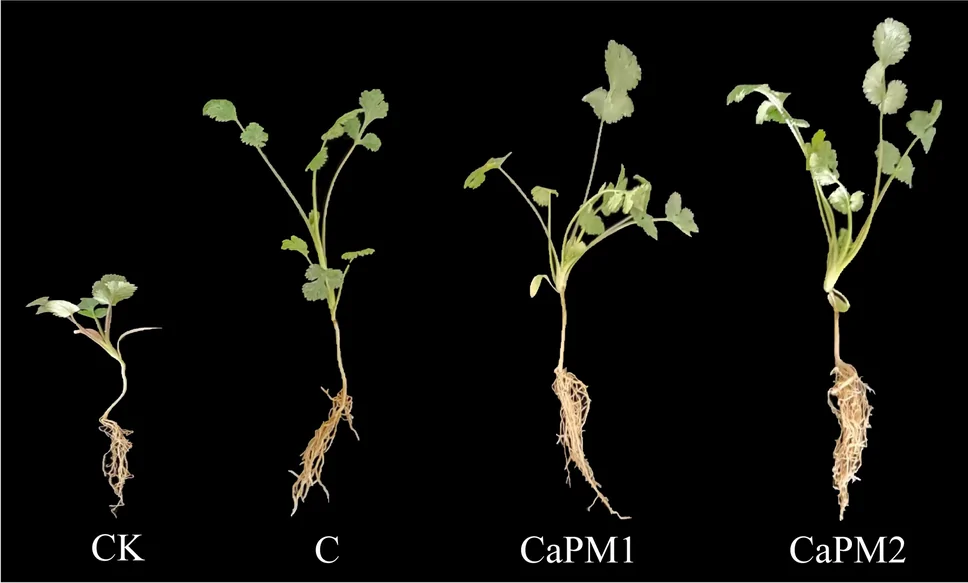
Cilantro planting

The samples harvested after 45 d of cilantro planting are shown in Fig. 5. Table 2 shows the growth and nutritional indices of potted cilantro. The plant moisture content of cilantro did not vary much between treatments, remaining in the range of 88.25 to 90.64%. The plant weight of cilantro with the addition of organic fertilizer was significantly better than that of CK. Among all treatments, the plant weight of CaPM2 was significantly better than the other treatment groups (*p* < 0.05). The CaPM2 treatment group had the highest plant height and the most extended root length of 14.41 cm and 13.63 cm, respectively. The chlorophyll contents of CK, C, CaPM1, and CaPM2 were 26.17 SPAD, 38.47 SPAD, 41.70 SPAD, and 46.79 SPAD, respectively, with CaPM2 having the highest chlorophyll content, significantly higher than CK and other treatment groups (*p* < 0.05). CaPM1 and CaPM2 root viability coefficients were significantly higher than the other groups (*p* < 0.05), up to 199.19 μg·g^−1^·h-^1^ and 187.24 μg·g^−1^·h^−1^, respectively. The highest vitamin C content of cilantro was CaPM2, which was significantly higher than CK (*p* < 0.05) and 302.20 mg·kg^−1^ more elevated than CK. The highest soluble sugar content and soluble protein content of cilantro were both in CaPM2 with 3.98 mg·g^−1^ and 22.99 mg·g^−1^, respectively, which were significantly higher than CK and other treatment groups (*p* < 0.05).

**Table 2:** Cilantro growth indicators and nutritional indicators

Treatment	CK	C	CaPM1	CaPM2
Plant moisture content (%)	90.64 ± 2.17^a^	90.34 ± 0.48^a^	89.83 ± 0.65^a^	88.25 ± 1.36^a^
Plant weight (g)	0.36 ± 0.23^c^	1.13 ± 0.07^b^	1.38 ± 0.09^b^	2.48 ± 0.08^a^
Plant height (cm)	7.08 ± 0.12^c^	12.57 ± 0.57^b^	13.33 ± 0.47^ab^	14.41 ± 0.45^a^
Root length (cm)	7.22 ± 3.19^b^	9.08 ± 0.33^ab^	13.53 ± 0.85^a^	13.63 ± 1.7^a^
Chlorophyll (SPAD)	26.17 ± 0.58^c^	38.47 ± 1.17^b^	41.7 ± 1.37^b^	46.79 ± 2.65^a^
Root vigor coefficient (μg·g^−1^·h^−1^)	109.75 ± 0.99^c^	154.67 ± 11.72^b^	189.19 ± 7.23^a^	187.24 ± 9.06^a^
Vitamin C (mg·kg^−1^)	820.58 ± 1.29^b^	838.97 ± 6.45^b^	1001.24 ± 119.12^ab^	1122.78 ± 62.29^a^
Soluble sugars (mg·g^−1^)	1.52 ± 0.07^c^	3.15 ± 0.21^b^	3.47 ± 0.11^b^	3.98 ± 0.13^a^
Soluble protein (mg·g^−1^)	11.58 ± 0.47^d^	16.18 ± 0.22^c^	19.1 ± 0.67^b^	22.99 ± 0.79^a^

Note: three replicates were set up for each treatment

Table 3 shows the potting soil's physicochemical properties after applying different treatments of organic fertilizers for growing cilantro. The soil pH of treatment C, CaPM1, and CaPM2 with organic fertilizer application was 7.20, 6.77, and 6.43, respectively, higher than that of CK (pH = 6.30). The potted soil OM content of C, CaPM1, and CaPM2 were all significantly elevated (*p* < 0.05) compared to CK, with the highest content of 45.39 g·kg^−1^ in the CaPM2 treatment. There was no significant difference in TN in each treatment group. TP and TK of organic fertilizer treatment were significantly better than CK (*p* < 0.05). AN is the most easily absorbed nitrogen in plants. The AN content of C, CaPM1, and CaPM2 are 13.62 mg·kg^−1^, 15.60 mg·kg^−1^, and 16.57 mg·kg^−1^, respectively. CaPM2 was significantly higher than CK and other treatments (*p* < 0.05). The highest content of AP and AK in soil was CaPM2 treatment, which was 22.27 mg·kg^−1^ and 1.29 mg·kg^−1^, respectively, which was significantly (*p* < 0.05) higher than CK and other treatment groups.

**Table 3:** Potting soil physicochemical properties

Treatment	CK	C	CaPM1	CaPM2
pH	6.3 ± 0.19^c^	7.2 ± 0.03^a^	6.77 ± 0.16^b^	6.43 ± 0.09^c^
Organic matter (g·kg^−1^)	30.75 ± 0.03^c^	40.84 ± 0.79^b^	44.45 ± 0.06^a^	45.39 ± 1.33^a^
Total phosphorus (mg·kg^−1^)	666.11 ± 185.95^b^	1025.45 ± 46.95^a^	1053.24 ± 36.3^a^	1138.03 ± 4.63^a^
Total potassium (g·kg^−1^)	2.48 ± 0.08^c^	3.37 ± 0.28^b^	3.89 ± 0.17^a^	3.93 ± 0.11^a^
Available phosphorus (mg·kg^−1^)	5.24 ± 0.58^d^	16.88 ± 0.62^c^	19.71 ± 0.41^b^	22.27 ± 0.49^a^
Available potassium (g·kg^−1^)	0.15 ± 0.02^c^	0.93 ± 0.04^b^	1.03 ± 0.08^b^	1.29 ± 0.05^a^
Total nitrogen (g·kg^−1^)	2.06 ± 0.1^b^	2.15 ± 0.01^ab^	2.24 ± 0.04^ab^	2.35 ± 0.02^a^
Alkali-hydrolyzed nitrogen (mg·kg^−1^)	9.81 ± 0.02^d^	13.62 ± 0.55^c^	15.6 ± 0.27^b^	16.57 ± 0.27^a^

Note: three replicates were set up for each treatment

## Discussion

### Effect on the physicochemical properties of compost

The temperature is an important index to reflect the microbial activity in the composting (Sun et al. 2019). The thermophilic stage (> 50 ℃) of all treatments exceeded 10 days. The continuous high temperature could kill pathogens (Wang et al. 2017) and facilitate the decay of compost (Jiang et al. 2014). At the end of composting, all treatments had GI values above 50%, indicating that the compost was not toxic to plants (Shehata et al. 2019). OM was the material and energy source for microbial metabolism to produce heat to maintain the high temperature of composting (Sun et al. 2022). TN loss was less in both CaPM1 and CaPM2 than in C during TS to MS. Phosphate can act as a buffer to the pH of the compost by both the directly reduced pH and the combined production of NH_4_^+^, while also enhancing microbial activity (Shou et al. 2019; Zhang and Sun 2017). The composting process of CaPM1 (pH 7.64 ~ 7.87) and CaPM2 (pH 7.21 ~ 7.49) was maintained in a more stable pH range. The stable pH is beneficial to the growth of microorganisms and the transformation of substances in the compost, due to the favorable pH buffering effect of Ca(H_2_PO_4_)_2_. Effective maintenance of a stable pH range facilitates struvite precipitation and retains more ammonia (Liang et al. 2018). It has also been reported that free or dissolved Mg^2+^ also has the ability to promote the synthesis of struvite (Thant Zin and Kim 2021). Ca(H_2_PO_4_)_2_ and MgSO_4_ can allow PO_4_^3−^ and Mg^2+^ to bind NH_4_^+^ and generate struvite in the pH 7.0 ~ 8.0 (Jiang et al. 2016; Wang et al. 2010), reducing the conversion of NH_4_^+^ to NH_3_, reducing the loss of nitrogen, and increasing the stability of nitrogen. The increase in TP and TK in the compost was due to decreased organic carbon content. As the organic matter was degraded and the compost’s total weight decreased, the compost’s TP and TK content tended to increase (Jiang et al. 2019). The AP and AK contents were higher in both CaPM1 and CaPM2 than in C. Organic matter containing phosphorus and potassium is broken down during composting, releasing it as more active AP and AK (Maleki et al. 2023). AP and AK can be used directly and rapidly by plants or directly after transformation, and they are essential measures of the fertility of organic fertilizers. This result indicates that adding Ca(H_2_PO_4_)_2_ and MgSO_4_ in composting can reduce nitrogen loss, achieve the effect of nitrogen retention, reduce nutrient loss, and maintain high organic fertilizer efficiency.

### Effects on bacterial community structure and interrelationships within bacteria

Compared with the C, the relative abundance of *Firmicutes* and *Actinobacteria* in CaPM1 and CaPM2 increased. *Firmicutes* had excellent OM degradation ability and heated resistance (Zhang et al. 2018; Tran et al. 2021). *Actinomycetes* had the ability to produce lignocellulolytic enzymes that enhance OM decomposition (Zhao et al. 2016). The increase in the relative abundance of *Firmicutes* and *Actinomycetes* in CaPM1 and CaPM2 may have been the main reason for the decrease in OM. The relative abundance of the *Proteobacteria* in C, CaPM1, and CaPM2 was 44.75%, 30.11%, and 20.29%, respectively, and the relative abundance of the treatment group was reduced relative to the control group. Due to the significant inhibitory effect of high temperature on *Proteobacteria* (Shi et al. 2021), CaPM1 and CaPM2 entered the thermophilic stage during composting more than C, and the thermophilic stage was maintained for a longer period of time, so it may be the reason for the decrease in the relative abundance of *Proteobacteria*. *Proteobacteria* is one of the keys affecting NH_4_^+^-N in pig manure compost, and NH_4_^+^-N was negatively correlated with GI (Kong et al. 2022). At the same time, the addition of Ca(H_2_PO_4_)_2_ and MgSO_4_ in the treatment increased a series of OM degrading bacteria, promoting the conversion of compost organic matter to humus and improving the degree of compost maturation. These may be responsible for the significantly higher GI values in CaPM1 and CaPM2 than in the control treatment. *Proteobacteria* are important denitrifying bacteria in the composting process and can contribute to N_2_O emissions (Zhong et al. 2020). The decrease in the relative abundance of *Proteobacteria* in CaPM1 and CaPM2 can reduce denitrification during the high temperature phase of composting and thus reduce nitrogen losses. The dominant genera in CaPM1 and CaPM2 were *Pseudoclavibacter*, *Thermobifida*, and *Bacillus*. *Thermobifida* and *Bacillus* are the main genera of organic matter degrading bacteria commonly found in the thermophilic stage of composting (Zhao et al. 2022; Wang et al. 2023a, b).

Ca^2+^ can regulate bacterial gene expression and biofilm synthesis (Kolodkin-Gal et al. 2023), activate the core functional microorganisms in compost, and increase the diversity and complexity of microorganisms (Wang et al. 2022). Mg^2+^ is an activator of enzymes, which can promote microbial metabolism (Vithani et al. 2020). Therefore, the joint action of Ca(H_2_PO_4_)_2_ and MgSO_4_ can promote the activity of microorganisms in compost. *Pseudoxanthomonas* was a significantly different microbial genus in CaPM1 (*p* < 0.05), which influences the basic nitrogen fixation community of NH_4_^+^-N transformation in compost (Wu et al. 2020). Among significantly different genera for CaPM2, *Pseudogracilibacillus* is associated with nitrogen conversion and the formation of nitrogenous humus at high temperatures (Li et al. 2020b; Yu et al. 2022). *Planifilum* secreted xylanase to accelerate the degradation of organic matter, and it was also related to the retention of nitrogen (Zhang et al. 2020). These bacterial genera associated with nitrogen transformation and nitrogen retention had significant variability in CaPM1 and CaPM2, which may have resulted in reduced nitrogen loss.

During the composting process, microorganisms transform substances inside the compost, which affects the physicochemical properties inside the compost. TN and AK were significantly positively correlated with *Saccharomonospora*, *Planifilum*, *Pseudoclavibacter*, and *Comamonas* (*p* < 0.05). The abundance of *Planifilum* was higher in CaPM1 and CaPM2 than in C, which may be one of the reasons for the higher TN content in CaPM1 and CaPM2 than in C during the thermophilic stage. Ca(H_2_PO_4_)_2_ and MgSO_4_ changed the bacterial community structure in compost, increased the bacteria related to cellulose degradation and nitrogen retention, and promoted the compost decay.

Co-occurrence network analysis showed that *Firmicutes* in the treatment group with the addition of Ca(H_2_PO_4_)_2_ and MgSO_4_ occupied a large number of nodes and edges in the network and played a stabilizing role in the overall microbial community. *Firmicutes* as the main compost thermophilic microorganisms increased in the thermophilic phase, which facilitated the degradation of OM and conversion to humus, accelerating compost humification and promoting compost maturation. Positive links and negative links in the networks represent mutual adaptation and competition in microbial ecology, respectively (Meng et al. 2023). Network analysis reflects the complex relationships between microorganisms, with more complex networks indicating more stable microbial communities (Song et al. 2023). The relationships among bacteria in all three networks showed positive and mutually beneficial relationships, which were favorable to composting. Fewer bacteria were involved in the negative links of the networks, which suggests less competition and a more balanced relationship between bacterial phyla. Both *Proteobacteria* and *Acidobacteriota* were involved in most of the negative linkages suggesting competition with species from other clades, due to the increased relative abundance of *Firmicutes* and *Actinobacteriota* in CaPM1 and CaPM2, which can also be seen from the network analyses as weakening the competitiveness of *Proteobacteria* and *Acidobacteriota*, which may also explain the decrease in the relative fractions of *Proteobacteria* and *Acidobacteriota*. Compared with C, there were more nodes and edges in the CaPM1 and CaPM2 networks, which suggests that the addition of Ca(H_2_PO_4_)_2_ and MgSO_4_ in composting activated more key bacterial species while enhancing microbial interactions and forming more complex network relationships. The complex network relationships resulted in a more stable microbial community structure. The addition of Ca(H_2_PO_4_)_2_ and MgSO_4_ to the compost improved the interaction of the compost bacterial community.

### Effects on nitrogen metabolism genes and the relationship between nitrogen metabolism genes and physicochemical properties

K00367 (*nirB*) and K00372 (*nasA*) are genes that regulate assimilatory ferredoxin-nitrate reductase and assimilatory nitrate reductase catalytic subunit enzymes, respectively. K03385 (*nrfA*) regulates the expression of nitrite reductase. In dissimilatory nitrate reduction, nitrite reductase converts nitrite to ammonia. The two enzymes are involved in the conversion of nitrate to nitrite in assimilatory nitrate reduction. In both CaPM1 and CaPM2, *nirB* and *nasA* are down-regulated, which indicates that the treatment groups of Ca(H_2_PO_4_)_2_ and MgSO_4_ can reduce the production of NO_2_^−^-N. Meanwhile, *nrfA* in CaPM2 is up-regulated to promote the conversion of nitrite into ammonia in the compost. The compost with 1.5% Ca(H_2_PO_4_)_2_ and 3% MgSO_4_ additions can not only reduce the production of NO_2_^−^-N but also promote the conversion of NO_2_^−^-N to NH_4_^+^-N and finally reduce the accumulation of NO_2_^−^-N in the compost. The rate-limiting step of nitrification is carried out by the involvement of ammonia monooxygenase subunit A enzymes, the level of which is regulated by K10944 (*amoA*) (Kuypers et al. 2018). In CaPM1 and CaPM2, *amoA* was regulated at a lower level than C. It was due to the abundance of *amoA* is affected by the temperature of the compost (Meng et al. 2020), and the high temperatures sustained during the thermophilic stage in CaPM1 and CaPM2 reduce the abundance of *amoA*, which may be explained by the reduction of nitrification in the compost by Ca(H_2_PO_4_)_2_ and MgSO_4_. Although K02586 (*nifD*) was down-regulated in nitrogen fixation with the addition of Ca(H_2_PO_4_)_2_ and MgSO_4_, K00260 (*gudB*) and K00262 (*gdhA*) were significantly up-regulated in the process of interconversion between NH_3_ and L-glutamate, and the conversion of NH_3_ to L-glutamate increased the stability of nitrogen. Changes in the abundance of key nitrogen metabolism genes showed that changes in the abundance of *nirB*, *nasA*, and *nrfA* in the treatment groups with the addition of Ca(H_2_PO_4_)_2_ and MgSO_4_ reduced the accumulation of nitroso-nitrogen and also reduced the denitrification process. At the same time, despite the reduction of genes associated with nitrogen fixation, the conversion of NH_3_ into nitrogenous organic compounds was promoted, increasing the stability of nitrogen.

Analysis by mantel test revealed a high degree of similarity between the correlation of compost additives with the physicochemical properties of compost and the correlation of nitrogen metabolism genes with the physicochemical properties of compost. Addition of additives to compost affects the physicochemical properties of compost, which in turn affects the nitrogen metabolism genes in the thermophilic stage of compost. Temperature, pH, and OM content were significantly correlated with nitrogen metabolism gene abundance (*p* < 0.01), which probably due to the fact that higher temperature and OM content affect the growth of nitrogen metabolism bacteria and thus the efficiency of nitrogen metabolism (Liu et al. 2020; Huang et al. 2022). High temperatures inhibit the growth of denitrifying bacteria (Xie et al. 2023; Zhao et al. 2020), and it has also been reported that PO_4_^3−^ increases the NO_x_-N content in compost, which also limits denitrification in compost (Elfadil et al. 2020). The addition of Ca(H_2_PO_4_)_2_ and MgSO_4_ to the treatment groups not only prolonged the high temperature period of the compost but also increased the concentration of PO_4_^3−^, which are most likely responsible for the down-regulation of denitrification-related genes in CaPM1 and CaPM2. Mg^2+^ can act as a cofactor for glutamate dehydrogenase to facilitate the catalytic reaction to take place (Liu and Birsoy 2023), and glutamate dehydrogenase is regulated by *gudA* and *gudB* (Ge et al. 2022). Added Mg^2+^ in CaPM1 and CaPM2, together with *gudA* and *gudB*, which are at up-regulated levels, facilitates the conversion of ammonia to L-glutamate in compost. Ca(H_2_PO_4_)_2_ and MgSO_4_ can affect the nitrogen metabolism genes by indirectly influencing the physicochemical properties of the compost affecting the nitrogen-converting bacteria and thus the nitrogen metabolism genes, as well as by direct effects of the nitrogen metabolism genes through PO_4_^3−^ and Mg^2+^.

### Effects on potted cilantro and the soil

Ca(H_2_PO_4_)_2_ and MgSO_4_ promoted the decay of compost and reduced the loss of nitrogen. The compost products obtained from composting enhanced the weight, root length, and plant height of cilantro and increased the yield of cilantro. Mg^2+^ was a vital component in the synthesis of chlorophyll. The organic fertilizer contained Mg^2+^, which could promote the synthesis of chlorophyll in cilantro, so the chlorophyll content of cilantro in CaPM1 and CaPM2 was higher than that in CK and C. By comparing each growth and nutrient index of potted cilantro, the compost product added to the CaPM2 treatment group promoted the growth and quality of cilantro. At the same time, the comparison of each physicochemical index of the potted soil showed that the CaPM2 treatment promoted soil fertility.

In conclusion, Ca(H_2_PO_4_)_2_ and MgSO_4_ increased the content of TN, AN, AP, and AK in the compost. Meanwhile, Ca(H_2_PO_4_)_2_ and MgSO_4_ changed the composition of bacterial community in the thermophilic stage of the compost and activated the bacteria associated with nitrogen retention. Ca(H_2_PO_4_)_2_ and MgSO_4_ also increased the microbial network complexity. These results contributed to compost maturity. The treatment groups with the addition of Ca(H_2_PO_4_)_2_ and MgSO_4_ reduced the accumulation of nitrite and the process of denitrification and increased the stability of nitrogen. Ca(H_2_PO_4_)_2_ and MgSO_4_ can affect the nitrogen metabolism genes by indirectly influencing the physicochemical properties of the compost affecting the nitrogen-converting bacteria and thus the nitrogen metabolism genes, as well as by direct effects of the nitrogen metabolism genes through PO_4_^3−^ and Mg^2+^. Composting with 1.5% Ca(H_2_PO_4_)_2_ + 3% MgSO_4_ produced the compost product that improved the growth yield and nutrient content of cilantro and increased the fertility of the soil. Ca(H_2_PO_4_)_2_ and MgSO_4_ reduce the loss of nitrogen from compost, activates nitrogen-related bacteria and genes in the thermophilic phase of composting, and improves the fertilizer efficiency of compost products.

## Data Availability

The data and material of this manuscript are available.
